# Parotitis—Mumps

**Published:** 1887-06

**Authors:** 


### Parotitis—Mumps.

I shall now speak of parotitis, an acute, contagious disease. It is oftentimes not preceded by premonitory symptoms. In many cases the first symptom will be soreness at the angle of the jaw and slight swelling of the parotid, but there will be little or no febrile action. The swelling of this disease occurs just below the lobe of the ear, pushing the lower lobe upwards, and the swelling extending on to the cheek as well, and on account of this swelling about the angle of the jaw mastication, or any movement of the jaw causes pain. The swelling reaches its height usually in about three or four days, then remains stationary and gradually subsides, so that by the sixth, or at most, the tenth day, it has disappeared. Mumps usually occur first upon one side (as a rule, the left). The parotid gland is the seat of the malady, then within from one to three days the opposite gland becomes inflamed. If only one gland is affected, then the other may be the seat of a subsequent attack. Occasionally the swelling disappears and metastasis occurs (in the male) in the testes, the epididymis or tunica vaginalis; while in the female it may be found in the labia majora, or ovaries, or mammæ. Sometimes metastasis may occur without the subsidence of the swelling of the parotid gland.

The disease can scarcely be mistaken for any other, for it pushes the lower lobe of the ear upwards; it projects upon the cheek; it is different from a swelling of the lymphatic or cervical glands; it is not so hard, indurated, and unyielding. We may have a swelling of the lymphatic gland in diphtheria, which may closely resemble a swelling of the parotid gland in mumps, but if you inspect the throat you will find the diphtheritic membrane characteristic of diphtheria. In a case of uncomplicated parotitis the pharnyx remains natural; there is no inflammation or swelling of the tonsils. Sometimes we may have a swelling of the lymphatic gland from a decayed molar tooth, but inspection of the mouth will help you in your diagnosis.

The prognosis is always favorable; it is simply a disease unattended by danger.

The treatment will consist of some diaphoretic mixture, using an opiate to control the pain, allowing the patient a little rest, local applications, etc. Usually oil will be sufficient, frequently applied to the swollen gland and covered with cotton or wool, or we may use flannels saturated with sweet or camphorated oil and apply it directly over the swollen and inflammed gland. It is well to protect the gland by means of cotton batting or a pad of some kind; in some cases it is prudent to apply some slight counter-irritant, as camphorated oil; we may give occasionally a

Dover's powder, especially if pain is present, to give the child rest. If metastasis occurs, this would be treated accordingly; warm poultices should be applied, and a very good application will be the following: Take of aconite leaves, one ounce; muriate of ammonia, one ounce. Place this mixture in a quart of water and steep it for ten or fifteen minutes, make a tea, then make a poultice of flaxseed meal, using this tea to make the poultice instead of water. This tea should be made hot and incorporated with the flaxseed, then it should be applied to the swollen portion. This, I think, will be all the treatment indicated in this disease.

